# The Florence Emotional Eating Drive (FEED): a validation study of a self-report questionnaire for emotional eating

**DOI:** 10.1007/s40519-021-01216-2

**Published:** 2021-05-27

**Authors:** Emanuele Cassioli, Enrico Calderani, Giulia Fioravanti, Lisa Lazzeretti, Carlo Maria Rotella, Eleonora Rossi, Valdo Ricca, Edoardo Mannucci, Francesco Rotella

**Affiliations:** 1 Psychiatry Unit, Department of Health Sciences, University of Florence, Largo Brambilla 3, 50134 Florence, Italy; 2 Department of Health Sciences, Psychology and Psychiatry Unit, University of Florence, via di San Salvi 12, 50100 Florence, Italy; 3 Department of Biomedical Experimental and Clinical Sciences, SOD Diabetology, University of Florence and Careggi University Hospital, Florence, Italy; 4 University of Florence and Careggi Teaching Hospital, Largo Brambilla 3, 50134 Florence, Italy; 5 Psychiatry Unit, University of Florence and Careggi Teaching Hospital, Largo Brambilla 3, 50134 Florence, Italy

**Keywords:** Emotional eating, Eating disorders, Obesity, Emotions, Type 2 diabetes

## Abstract

**Purpose:**

Emotional eating is a trans-diagnostic dimension in eating disorders and is present in many other conditions that could affect eating attitudes. At present, there is no instrument that measures emotional eating evaluating both the intensity and the frequency of emotion-induced desire to eat. The aim of the study was the validation of the Florence Emotional Eating Drive (FEED).

**Methods:**

A sample of healthy volunteers was initially enrolled to explore internal consistency and test–retest reliability. The Emotional Eating Scale (EES), Eating Disorders Evaluation-Questionnaire (EDE-Q), Binge Eating Scale (BES) and Symptom Checklist-90 (SCL-90-R), together with the final version of FEED, were administered to a clinical sample composed by patients with eating disorders, obesity, and type 2 diabetes, to explore the underlying structure of the questionnaire and verify its validity.

**Results:**

FEED showed excellent internal consistency (Cronbach’s alpha = 0.96) and test–retest reliability (*r* = 0.93). FEED scores were higher in patients with BN and BED than in AN patients, negatively correlated with age and positively with BES and EES. Multiple regression analysis showed that FEED, but not EES, was independently associated with SCL-90-R and EDE-Q scores.

**Conclusion:**

FEED internal consistency and test–retest reliability were excellent. The addition of specific questions on the frequency of behaviours led to a better component structure and robustness compared to EES. A tool that reliably and specifically assesses eating behaviours driven by emotional states may be extremely useful in clinical settings.

**Level of evidence:**

Level V, cross-sectional study.

**Supplementary Information:**

The online version contains supplementary material available at 10.1007/s40519-021-01216-2.

## Introduction

The impact of biology, society, culture and environment on emotions has been often debated [1–3]. Some researchers think that emotions are universal constructs, mostly biologically determined [4–6], whereas others consider culture, society and environment as important as biology. In fact, environmental factors affect the way in which emotions are felt and expressed by providing a paradigm of how people should feel and the degree of appropriate emotional expression in a given context [7–9]. The inability to properly modulate emotional responses is known as emotion dysregulation, which is commonly associated with general psychopathology [10]. Given that emotion dysregulation is associated with core psychopathological and behavioural features of eating disorders (EDs) [11], and that food can represent a maladaptive strategy of emotional regulation [12], some authors consider the impaired cognitive capacity to process and regulate emotions as a key feature of EDs [11, 13, 14]. For this reason, emotional eating, which is the desire to eat in response to different emotional states [15], was suggested as a trans-diagnostic feature in EDs, regardless of the presence of binge eating or overeating behaviours [16–18]. In fact, it has been reported that emotional eating is present in patients with Anorexia Nervosa (AN) [17], obesity [19] and diabetes [20].

The DSM-5 [21] stated that psychiatric nosography “should accommodate ways to introduce dimensional approaches to mental disorders, including dimensions that cut across current categories. Such an approach should permit a more accurate description of patient presentations and increase the validity of a diagnosis”; such approaches “will likely supplement or supersede current categorical approaches in coming years”. Following these statements, the construct of emotional eating could assume particular relevance, as it could represent a common thread through many different conditions in the light of emotion dysregulation. Furthermore, even if a specific emotion can lead to a significative change in cognitive or behavioural pattern, it has been proposed that an increased or decreased frequency in experiencing emotions could determine the same effect [10]: for this reason, investigating the frequency with which emotions occur could be important in characterizing emotional eating. Furthermore, the data on the frequency of emotion is fundamental so that the assessment of emotional eating can guide any clinical intervention: if a patient reports overeating in the presence of an emotion that almost never occurs, it may not be clinically relevant (and advisable to intervene). To the best of our knowledge, the Emotional Eating Scale (EES) [15] is the most widely used validated test that specifically investigates the emotional eating dimension across diagnostic entities. The EES explores the patients’ perception of the impact of different emotions on their desire to eat; however, it does not provide any indication on the frequency with which the emotions affecting eating behaviours occur, and therefore it does not appropriately reflect the clinical relevance of the symptom. Other validated questionnaires assessing emotional eating are not commonly used in clinical settings [22–27], and, in most cases, explore a range of disturbances wider than emotional eating per se (e.g. the Emotional Eater Questionnaire—EEQ [24] was designed for the assessment of emotional eating in subjects with obesity and assesses craving and binge attitudes for specific foods).

To have a more reliable and clinically useful assessment of emotional eating, both intensity and frequency of occurrence should be explored in a single questionnaire. Such an approach has been previously adopted for assessing the quality of life in patients with obesity and type 1 diabetes [28, 29]. In the present study, we aim to demonstrate the validity and reliability of the Florence Emotional Eating Drive (FEED) questionnaire for the assessment of emotional eating, exploring the occurrence of specific emotions together with the association between such emotions and the urge to eat.

## Methods

The test was developed on the basis of a pre-existing questionnaire, the EES [15], which explores the patients’ perception of the impact of different emotions on their desire to eat; however, it does not provide any indication of the frequency with which the emotions affecting eating behaviour actually occur. In the present questionnaire, each item of the EES was completed with a second question, exploring the frequency of the emotion: details regarding the scale and the scoring method are reported below.

Validation studies, after approval by the local Ethical Board, were performed in two distinct samples of participants, who all provided their informed consent:

**Sample 1.** A sample of 100 healthy volunteers aged between 18 and 65 years was enrolled using convenience snowball sampling (among medical students and their relatives and friends); subjects reporting a lifetime occurrence of any mental disorder were excluded, as well as illiterate participants and those not fluent in Italian. Of the initial sample, 94 participants filled the questionnaire and were therefore included in the analysis. This group was asked to complete the first draft of the questionnaire. This initial draft consisted of all 25 items originally included in the EES, to each of which a corresponding Likert scale was added for measuring the frequency of that emotion (in addition to the urge to eat), for a total of 25 emotions each assessed with 2 sub-items. The first version of the questionnaire was reviewed by a panel of medical specialists in psychiatry for the initial assessment of face validity. Initial calculations of internal consistency were performed on this sample, using Cronbach’s alpha. Descriptive statistics were calculated for the total score and for each individual item (mean and standard deviation). The Pearson correlation coefficient was also computed for the association of each item with the total score (item-total correlation) and with the total score without the item itself. To be considered valid, a self-reported questionnaire should show satisfactory test–retest reliability, i.e. the test administered twice at a reasonably short time interval should provide approximately the same results. For this reason, a subgroup of 53 participants completed the questionnaire again after 2 weeks, and test–retest reliability was evaluated by calculating the intraclass correlation coefficient (ICC), using a single-measurement two-way mixed effects model. These analyses led to a further improvement of the questionnaire thanks to the removal of the items that showed unfavourable characteristics.

**Sample 2**. To verify concurrent, convergent and discriminant validity of FEED, the final version of the test was administered to a clinical sample of patients, composed of the following subgroups:

1. A consecutive series of patients with eating disorders (either anorexia nervosa [AN], bulimia nervosa [BN], or binge eating disorder [BED], following DSM-5 criteria) [21] attending the Outpatient Clinic for Eating Disorders of Careggi teaching hospital in Florence. Patients with bipolar disorder or psychotic disorders were excluded, as well as those aged < 18 or > 65 years, illiterate, or not fluent in Italian. Of the 110 patients invited, 100 provided their informed consent and were enrolled in the study. Of those enrolled, 37, 28, and 35 were affected by AN, BN, and BED, respectively.
2. A consecutive series of patients with obesity (body mass index > 30 kg/m^2^) aged 18–65 years attending the Obesity outpatient clinic of Careggi teaching hospital in Florence, excluding illiterate participants, those not fluent in Italian, and those who had previously undergone bariatric surgery. Patients with mental disorders (any axis I disorder, included eating disorders), as detected with a non-structured interview by a trained psychiatrist, which is part of the routine assessment of the Clinic, were excluded. Of the 90 invited, 82 accepted participation.
3. A consecutive series of patients with type 2 diabetes aged 18–65 years, attending the Diabetes outpatient clinic of Careggi teaching hospital in Florence, excluding illiterate participants, those not fluent in Italian, and those taking antipsychotic medication. Of the 60 invited, 52 accepted participation.

**Table 1:** FEED items characteristics in Sample 1

	Mean (*n* = 94)	SD (*n* = 94)	Item-total correlation (*r*)	Item-total correlation item dropped (*r*)	Retest ICC (*n* = 53)
Item 1—Resentful	1.13	1.88	0.57	0.54	0.89
Item 2—Discouraged	1.79	2.04	0.75	0.71	0.69
Item 3—Shaky	1.66	1.95	0.54	0.49	0.75
Item 4—Worn out	1.26	1.91	0.51	0.45	0.73
Item 5—Inadequate	1.26	1.81	0.49	0.46	0.71
Item 6—Excited	1.19	1.78	0.30	0.24	0.79
Item 7—Rebellious	0.77	1.44	0.55	0.47	0.71
Item 8—Blue	1.96	1.88	0.61	0.54	0.54
Item 9—Jittery	2.13	2.57	0.54	0.54	0.85
Item 10—Sad	2.11	1.86	0.41	0.36	0.58
Item 11—Uneasy	0.79	1.36	0.49	0.45	0.38
Item 12—Irritated	1.40	1.94	0.68	0.63	0.65
Item 13—Jealous	0.66	1.54	0.59	0.54	0.73
Item 14—Worried	2.40	2.36	0.64	0.61	0.91
Item 15—Frustrated	1.09	1.35	0.63	0.60	0.60
Item 16—Lonely	1.49	2.01	0.42	0.39	0.87
Item 17—Furious	0.91	1.55	0.61	0.59	0.72
Item 18—On edge	2.13	2.06	0.47	0.45	0.70
Item 19—Confused	0.87	1.40	0.45	0.38	0.60
Item 20—Nervous	2.47	2.55	0.70	0.68	0.63
Item 21—Angry	1.72	2.18	0.56	0.51	0.81
Item 22—Guilty	1.08	1.34	0.38	0.35	0.53
Item 23—Bored	1.89	2.18	0.39	0.34	0.81
Item 24—Helpless	0.25	0.73	0.30	0.21	0.42
Item 25—Upset	0.15	0.41	0.39	0.30	0.63
FEED Total Score	1.38	0.97			0.93

### Questionnaires

All patients of Sample 2 were administered the following questionnaires:

- FEED: the final version of FEED consisted of the 23 items that showed favourable psychometric properties in the preliminary analyses performed in Sample 1. Assessed emotions are the same as EES, minus “excited” (item 6 of EES) and “helpless” (item 24 of EES). Every emotion is evaluated on two different 0–4 Likert scales, both in terms of frequency (“How often do you feel…?”: “Never”, “A few times”, “Sometimes”, “Often”, “Always”) and corresponding urge to eat (“How strong is your drive for eating when you feel…?”: “No desire to eat”, “A small desire to eat”, “A moderate desire to eat”, “A strong urge to eat”, “An overwhelming urge to eat”). Assessed emotions are listed in the complete questionnaire, which is reported in the Supplementary Material. For each item, the frequency of the emotion and the corresponding drive to eat are combined to obtain a final item score on the basis of score ranks, as indicated in the scoring table (Table 1 of Supplementary Material). The scoring table was designed in such a way as to weigh the two information equally and to return a final item score on a ten-point rating scale (0–9), where zero corresponds to the absence of the urge to eat in the presence of that emotion or the absence of the emotion itself, and nine corresponds to an overwhelming urge to eat for an emotion that is always felt. The goal of the composite score was to create a measure that was closer to the clinical relevance of the symptom and, therefore, more useful in a clinical context: in two individuals who have the same propensity towards emotional eating, it would be expected that the one who is more frequently emotionally upset gets higher scores than one who is almost never upset.
- EES [15]: the questionnaire for measuring the drive towards emotional eating in the presence of an emotion, on which the initial development of the FEED was based; the evaluation of emotional eating is carried out without taking into consideration its frequency. A total of 25 emotions are covered by this scale, which provides a total score and 3 subscales for anger/frustration, anxiety and depression. The original validation study found acceptable psychometric characteristics (Cronbach’s alpha = 0.81; r_test–retest_ = 0.79, p < 0.001) [15]; however, it was widely criticized for the low sample size and the non-reproducibility of the factor analysis [30].
- Eating Disorder Examination Questionnaire (EDE-Q) [31]: this self-reported questionnaire consists of 28 items assessing the core psychopathological features of eating disorders, with a total score and 4 subscales: dietary restraint, eating concern, weight concern, and shape concern. EDE-Q showed excellent internal consistency (Cronbach’s alpha = 0.94) and test–retest reliability (Spearman’s rho = 0.80, *p* < 0.001) [32].
- Binge Eating Scale (BES) [33]: this questionnaire was proposed as a rapid screening instrument for BED in patients with obesity, and it examines both behavioural signs (eating large amounts of food) and feeling or cognition during a binge episode (loss of control, guilt, fear of being unable to stop eating) through 16 items. The scale showed good psychometric properties in validation studies (Cronbach’s alpha ≥ 0.85; r_test–retest_ = 0.87, *p* < 0.001) [34, 35].
- SCL-90-R: The Symptom Checklist-90 Revised [36] is a widely used psychometric instrument devoted to the identification of general psychopathologic distress, which demonstrated good psychometric properties (Cronbach’s alpha between 0.70 and 0.96) [37]. Although it provides multiple subscales relating to various psychopathological domains, only the total score was used for the purposes of this study (GSI: Global Severity Index).

### Statistical analysis

Means and standard deviations of scores of all psychometric tests were calculated in each patient group and compared between different diagnoses using Analysis of Variance (ANOVA), and post hoc analysis for statistically significant models was performed using Tukey’s test. Cronbach’s alpha was calculated for FEED. Principal Component factor Analysis (PCA) was used to extract the main subscales of the questionnaire. Suitability for PCA was assessed by inspecting the correlation matrix of all items and by means of Kaiser–Meyer–Olkin (KMO) measures and Bartlett’s test of sphericity. The number of factors to extract was determined using Horn’s parallel analysis. An oblique rotation (Promax) was applied to aid interpretability; a solution with an orthogonal rotation (Varimax) was also obtained.

The correlation of FEED with clinical and psychometric variables was explored by calculating Pearson correlation coefficients. To test the hypothesis that FEED scores presented a more robust association than EES with psychopathology measures, two multiple regression analyses were performed, in which FEED and EES were both entered as independent variables (with SCL-90-R and EDE-Q scores as dependents). This allowed to calculate adjusted (partial) regression coefficients, to verify which variable among FEED and EES retained a significant association with psychopathology measures, while adjusting for the effect of the other.

All analyses were performed using R version 4.0.2 [38], with the following packages: dplyr [39], GPArotation [40], psych [41], and stargazer [42].

## Results

**Sample 1.** Sample 1 consisted of 62 (66.0%) women and 32 (34.0%) men. The average age was 37.0 ± 13.2 years. The data obtained from the administration of the first version of FEED in Sample 1 are reported in Table 1. FEED had a high level of internal consistency, as determined by a Cronbach’s alpha of 0.89. All items showed good item-total correlation (r > 0.30), except items 6 (excited) and 24 (helpless) (Table 1). The item-total correlation of these items further decreased when the item itself was removed from the total score (Table 1). Given their poor overall psychometric properties, items 6 and 24 were removed from the final version of FEED (reported in the Supplementary Material).

The subsample that participated in the test–retest assessment consisted of 40 (75.5%) women and 13 (24.5%) men. FEED showed an excellent test–retest reliability, with a total score ICC > 0.90 (Table 1) and a test–retest correlation of *r* = 0.93. The test–retest ICC was satisfactory for all items, with a range of 0.38–0.91 (Table 1).

**Sample 2.** In this larger and more heterogeneous sample (*n* = 234), Cronbach’s alpha was 0.96. A PCA was run on the 23-question version of FEED. PCA was considered suitable, given the high inter-correlation between different items (all *r* > 0.30), an overall KMO measure of sampling adequacy of 0.95 (all individual KMO measures ≥ 0.90) and a statistically significant Bartlett’s test of sphericity (*p* < 0.001), indicating that the data was likely factorizable. Horn’s parallel analysis determined that the number of factors to be extracted was three; the three-component solution explained 58.0% of the total variance. Factor analyses with different rotation methods (Promax, Varimax) led to similar results, with the same final structure: all rotated components coefficients are reported in Table 2. Items with predominant loading on factor 1, 2, and 3 appeared to be mainly related to depression, anger, and anxiety, respectively. Strong intercorrelations between factors was observed: r_Depression–Anger_ = 0.76, *p* < 0.001; r_Depression-Anxiety_ = 0.79, *p* < 0.001; r_Anger–Anxiety_ = 0.75, *p* < 0.001.

**Table 2:** Rotated structure matrix for PCA of FEED, with Promax and Varimax rotations

	Promax rotation			Varimax rotation		
	Factor 1 Depression	Factor 2 Anger	Factor 3 Anxiety	Factor 1 Depression	Factor 2 Anger	Factor 3 Anxiety
Item 10—Uneasy	**0.84**	0.01	− 0.03	**0.75**	0.27	0.23
Item 21—Guilty	**0.83**	0.05	− 0.13	**0.70**	0.26	0.19
Item 5—Inadequate	**0.77**	− 0.19	0.23	**0.73**	0.17	0.35
Item 14—Frustrated	**0.60**	0.12	0.16	**0.64**	0.35	0.36
Item 23—Upset	**0.60**	0.18	− 0.09	**0.53**	0.31	0.20
Item 18—Confused	**0.47**	− 0.02	0.32	**0.51**	0.24	0.44
Item 2—Discouraged	**0.46**	0.05	0.37	**0.59**	0.35	0.45
Item 9—Sad	**0.45**	0.04	0.38	**0.57**	0.33	0.47
Item 15—Lonely	**0.37**	− 0.02	0.36	**0.47**	0.23	0.42
Item 16—Furious	0.19	**0.94**	− 0.29	0.35	**0.79**	0.11
Item 20—Angry	0.02	**0.82**	0.09	0.31	**0.78**	0.34
Item 11—Irritated	0.13	**0.71**	0.06	0.37	**0.70**	0.30
Item 6—Rebellious	− 0.26	**0.58**	0.20	0.03	**0.50**	0.26
Item 1—Resentful	0.29	**0.55**	− 0.04	0.40	**0.56**	0.23
Item 17—On edge	0.21	**0.47**	0.22	0.40	**0.55**	0.41
Item 8—Jittery	0.16	**0.45**	0.27	0.40	**0.56**	0.42
Item 19—Nervous	0.08	**0.45**	0.35	0.36	**0.56**	0.46
Item 7—Blue	0.04	0.02	**0.75**	0.33	0.31	**0.64**
Item 22—Bored	− 0.04	0.03	**0.74**	0.27	0.29	**0.60**
Item 3—Shaky	0.34	− 0.18	**0.63**	0.48	0.17	**0.58**
Item 13—Worried	0.06	0.16	**0.60**	0.34	0.39	**0.57**
Item 4—Worn out	0.10	0.09	**0.43**	0.30	0.26	**0.41**
Item 12—Jealous	− 0.08	0.20	**0.37**	0.12	0.29	**0.35**

Major loadings for each item are bolded

Total and subscale scores of FEED and other psychometric tests are summarized in Table 3. Scores of both FEED and EES were higher in patients with BN and, to a lesser extent, in those with BED (Table 3). Correlations of FEED scores with age, BMI and other psychometric tests are shown in Table 4. FEED scores showed a significant negative correlation with age and a positive correlation with BES and EES (Table 4). FEED also exhibited positive correlations with measures of general and ED-specific psychopathology and BMI (Table 4).

**Table 3:** Clinical and psychopathological characteristics of Sample 2, divided by diagnosis, together with comparisons between groups and post hoc tests

	AN (*n* = 37)	BN (*n* = 28)	BED (*n* = 35)	Diabetes (*n* = 52)	Obesity (*n* = 82)	*F*
Age (years)	25.68 ± 9.80^†,§,¶^	27.92 ± 7.67^§,¶^	42.37 ± 11.46^‡,§^	69.29 ± 11.30^¶^	48.06 ± 13.93	93.35***
BMI (kg/m^2^)	16.60 ± 2.13^†,‡,§,¶^	23.12 ± 5.00^§,¶^	45.41 ± 8.75^‡,§,¶^	29.72 ± 6.13^¶^	41.52 ± 6.23	129.64***
FEED						
Depression	1.71 ± 1.74^‡^	4.26 ± 2.48^§,¶^	2.45 ± 1.44^‡,§^	1.15 ± 1.50	1.60 ± 1.64	15.99***
Anger	1.53 ± 1.69^†,‡^	3.42 ± 2.27^§,¶^	2.86 ± 1.83^§^	1.67 ± 1.97	2.00 ± 1.91	5.69***
Anxiety	1.99 ± 1.72^‡^	3.82 ± 1.98^§,¶^	2.51 ± 1.69^‡,§^	1.35 ± 1.57	1.92 ± 1.67	9.73***
Total score	1.71 ± 1.56^‡^	3.84 ± 2.11^§,¶^	2.61 ± 1.36^‡,§^	1.38 ± 1.59	1.82 ± 1.61	11.53***
SCL-90-R						
Somatization	1.47 ± 0.94^§^	1.52 ± 0.75^§^	1.31 ± 0.75	0.94 ± 0.80	1.16 ± 0.73	3.75**
Obsessive–compulsive	1.80 ± 1.12^†,§,¶^	1.99 ± 0.96^§,¶^	1.22 ± 0.73^‡^	0.89 ± 0.77	0.90 ± 0.70	14.90***
Interpersonal sensitivity	1.62 ± 1.10^§,¶^	1.99 ± 0.99^§,¶^	1.18 ± 0.90^‡,§^	0.62 ± 0.54	0.87 ± 0.80	16.38***
Depression	2.04 ± 1.06^†,§,¶^	2.06 ± 1.01^§,¶^	1.20 ± 0.79^‡^	0.86 ± 0.64	1.00 ± 0.76	19.12***
Anxiety	1.58 ± 1.06^†,§,¶^	1.69 ± 0.91^§,¶^	0.91 ± 0.76^‡^	0.68 ± 0.59	0.70 ± 0.59	16.33***
Hostility	1.14 ± 0.94^§,¶^	1.11 ± 0.92	0.81 ± 0.73	0.62 ± 0.51	0.68 ± 0.64	4.49**
Phobic anxiety	0.79 ± 0.79^§,¶^	1.02 ± 0.86^§,¶^	0.56 ± 0.67^‡^	0.21 ± 0.24	0.28 ± 0.45	13.37***
Paranoid ideation	1.55 ± 1.13^§,¶^	1.78 ± 0.96^§,¶^	1.03 ± 0.81^‡^	0.71 ± 0.57	0.76 ± 0.66	13.86***
Psychoticism	1.22 ± 1.25^†,§,¶^	1.36 ± 0.83^§,¶^	0.65 ± 0.69^‡^	0.42 ± 0.36	0.47 ± 0.51	13.95***
GSI	1.53 ± 0.95^†,§,¶^	1.67 ± 0.79^§,¶^	1.04 ± 0.65^‡^	0.71 ± 0.50	0.82 ± 0.55	15.96***
EDE-Q						
Dietary restraint	3.26 ± 1.84^†,§,¶^	3.33 ± 1.69^§,¶^	1.94 ± 1.87^‡,§^	1.00 ± 1.10	1.27 ± 1.46	20.08***
Eating concern	2.60 ± 1.85^§,¶^	3.17 ± 1.46^§,¶^	2.43 ± 1.34^§,¶^	0.65 ± 0.87	1.28 ± 1.26	24.37***
Weight concern	3.04 ± 1.79^§^	3.84 ± 1.49^§,¶^	3.56 ± 1.03^§^	1.31 ± 1.22^¶^	2.81 ± 1.32	22.04***
Shape concern	3.50 ± 1.86^§^	4.23 ± 1.44^§,¶^	4.18 ± 0.96^§,¶^	1.47 ± 1.49^¶^	3.29 ± 1.46	25.35***
Total score	3.10 ± 1.71^§,¶^	3.64 ± 1.29^§,¶^	3.03 ± 0.98^§,¶^	1.11 ± 1.05^¶^	2.14 ± 1.09	27.89***
BES total score	14.19 ± 7.83^‡,§^	27.32 ± 11.52^§,¶^	18.40 ± 8.07^‡,§,¶^	5.65 ± 7.13^¶^	11.53 ± 8.39	32.46***
EES						
Anger	1.60 ± 0.59^†,‡^	2.88 ± 1.06^§,¶^	2.57 ± 0.78^§,¶^	1.71 ± 0.87	1.72 ± 0.98	15.60***
Anxiety	1.71 ± 0.67^†,‡^	2.80 ± 0.93^§,¶^	2.59 ± 0.96^§,¶^	1.77 ± 0.83	1.73 ± 0.89	13.34***
Depression	2.05 ± 0.87^†,‡^	2.94 ± 1.09^§,¶^	2.76 ± 0.86^§,¶^	1.85 ± 0.78	1.83 ± 1.10	11.41***
Total	1.73 ± 0.60^†,‡^	2.86 ± 0.97^§,¶^	2.61 ± 0.73^§,¶^	1.76 ± 0.80	1.75 ± 0.93	15.82***

*p < 0.05, **p < 0.01, ***p < 0.001

^†^Different from BED

^‡^Different from BN

^§^Different from Diabetes

^¶^Different from Obese

**Table 4:** Clinical and psychopathological correlates of FEED scores

	FEED Depression	FEED Anger	FEED Anxiety	FEED Total score
Age	− 0.31***	− 0.17*	− 0.31***	− 0.28***
BMI	0.07	0.19**	0.12	0.14*
SCL-90-R				
Somatization	0.39***	0.36***	0.37***	0.40***
Obsessive–compulsive	0.52***	0.43***	0.51***	0.52***
Interpersonal sensitivity	0.53***	0.37***	0.44***	0.49***
Depression	0.54***	0.41***	0.47***	0.51***
Anxiety	0.51***	0.40***	0.44***	0.49***
Hostility	0.38***	0.40***	0.31***	0.40***
Phobic anxiety	0.50***	0.33***	0.41***	0.45***
Paranoid ideation	0.43***	0.29***	0.38***	0.39***
Psychoticism	0.51***	0.35***	0.45***	0.47***
GSI	0.55***	0.43***	0.49***	0.53***
EDE-Q				
Dietary restraint	0.24***	0.16*	0.19**	0.21**
Eating concern	0.49***	0.37***	0.35***	0.45***
Weight concern	0.44***	0.39***	0.34***	0.43***
Shape concern	0.41***	0.38***	0.33***	0.41***
Total score	0.45***	0.37***	0.35***	0.43***
BES total score	0.69***	0.55***	0.55***	0.65***
EES				
Anger	0.70***	0.74***	0.67***	0.77***
Anxiety	0.67***	0.72***	0.63***	0.74***
Depression	0.61***	0.61***	0.65***	0.67***
Total	0.71***	0.75***	0.69***	0.78***

*p < 0.05, **p < 0.01, ***p < 0.001

Finally, multiple regression analysis showed that FEED, but not EES, was independently associated with both SCL-90-R (b_FEED_ = 0.21, *p* < 0.001; b_EES_ = 0.01, *p* = 0.97) and EDE-Q (b_FEED_ = 0.27, *p* = 0.002; b_EES_ = 0.19, *p* = 0.26) total scores.

## Discussion

The FEED questionnaire is the first instrument designed to assess the impact of emotions on eating behaviours, taking into account both the intensity and the frequency of emotion-induced desire to eat.

Test–retest reliability of FEED, which was assessed in Sample 1, was satisfactory; this confirmed that the questions were unambiguous. Considering internal consistency, the final value of Cronbach’s alpha was greater than 0.95, confirming the excellent coherence of the questionnaire. Moreover, FEED showed good construct validity, with distinctive elements compared to the EES. In clinical samples, as expected, scores of FEED were higher in patients with disorders characterized by loss of control over eating, i.e. BN and BED, and lower in those with type 2 diabetes, the majority of whom are not supposed to be affected by relevant eating disorder symptoms [43, 44]. Consequently, these results corroborate the concurrent validity of the questionnaire.

FEED scores showed a significant correlation with SCL-90-R and EDE-Q scores: this is not surprising, considering the known link between emotional eating and psychopathology, observed both in the general population [19] and patients suffering from EDs [45]. Furthermore, eating disorder symptoms tend to be more frequent in participants with any clinically relevant psychiatric condition, and eating disorders display relevant psychiatric comorbidities [46, 47]. Moreover, these correlates were also maintained in multivariate analyses where EES was entered as a covariate. Conversely, in multivariate models, EES scores did not retain a statistically significant association with psychopathology. These findings, together with the correlation between FEED and BES scores, confirm the convergent validity of the questionnaire, and support the hypothesis that FEED provides a score that is more indicative of the real dysfunctional nature of emotional eating for the individual, and more clinically relevant than EES, thanks to the composite scores that included both frequency and intensity of emotional eating.

Compared to the EES, the final version of FEED showed better psychometric properties, with higher internal consistency (alpha_FEED_ = 0.96 vs alpha_EES_ = 0.81) and two-week test–retest reliability (r_FEED_ = 0.93 vs r_EES_ = 0.79). Notably, FEED scores seem to discriminate across samples with different severity of eating psychopathology and loss of control to a greater extent than EES. Furthermore, the initially validated version of the EES (which is used to date) has a widely criticized subscale structure, evaluated on less than 50 subjects [15], and which could not be replicated in subsequent studies [30]; the poor dimensional validity led to the erroneous attribution of items on depressive emotions such as “Guilty”, “Discouraged” and “Inadequate” to the “Anger/Frustration” subscale. Conversely, FEED underwent a more rigorous validation procedure, which showed a more consistent component structure.

A test that specifically and reliably assesses emotional eating is potentially useful in many clinical settings. In patients seeking treatment either for obesity or eating disorders, the assessment of emotional eating is relevant for the characterization of patient profiles. Emotional eating, if properly identified and measured, can be an important therapeutic target to be dealt with through specific psychotherapeutic interventions. Theoretically, FEED could also be used to measure the efficacy of specific treatments for emotional eating, although its sensitivity to change after a therapeutic intervention was not assessed in the present study. In addition, FEED could also be used for identifying patients with emotional eating among those with non-psychiatric conditions potentially affected by eating behaviour, such as type 2 diabetes and other metabolic diseases. Further studies are needed to explore the potential use of FEED as a diagnostic instrument and/or as a measure of treatment outcome in clinical settings.

## Strength and limits

The initial exploratory analyses for the development of the final version of the questionnaire and the final validation analyses were carried out on two different samples to reduce the probability of overfitting. Analyses were performed on several different diagnostic categories, allowing for the use of the questionnaire in a broad clinical population. Moreover, FEED underwent a stricter validation procedure than the scale on which it was originally based (EES) and showed better psychometric characteristics.

The questionnaire was originally developed and validated in Italian. A limitation of the present study is represented by the small size of the male samples. Further research is needed to confirm the validity of this instrument in the male gender. In addition, the samples included only participants aged 18–65 years, meaning that FEED is not validated for use in paediatric or geriatric age.

## What is already known on this subject?

Emotional eating is a trans-diagnostic dimension in eating disorders and is present in many other conditions that could affect eating attitudes. At present, there is no instrument that measures emotional eating evaluating both the intensity and the frequency of emotion-induced desire to eat.

## What this study adds?

This study reports the development and validation procedure of a new questionnaire for the assessment of emotional eating, the Florence Emotional Eating Drive (FEED), which uses composite scores based on both the intensity and the frequency of emotion-induced desire to eat.

## Supplementary Information

Below is the link to the electronic supplementary material.

Supplementary file1 (DOCX 23 KB)

## Data Availability

Research data are not shared.
