# Supplementary material for: The Florence Emotional Eating Drive (FEED): a validation study of a self-report questionnaire for emotional eating
Source: Eat Weight Disord. 2021 May 27;27(2):751–9. doi: 10.1007/s40519-021-01216-2 (PMC8933357; doi:10.1007/s40519-021-01216-2)
Supplement: Supplementary file 1 — Supplementary file1 (DOCX 23 KB) [file 40519_2021_1216_MOESM1_ESM.docx]

**The Florence Emotional Eating Drive (FEED): a validation study of a self-report questionnaire for Emotional Eating**

*Eating and Weight Disorders - Studies on Anorexia, Bulimia and Obesity*

Emanuele Cassioli, MD^1^; Enrico Calderani, MD^1^; Giulia Fioravanti, PhD^2^; Lisa Lazzeretti, MD^1^; Carlo Maria Rotella, MD^3^; Eleonora Rossi, MD^1^; Valdo Ricca, MD^1^; Edoardo Mannucci, MD^4^; Francesco Rotella, PhD, MD^5^.

^1^ Psychiatry Unit, Department of Health Sciences, University of Florence, Largo Brambilla 3, 50134, Florence, Italy.

^2^ Department of Health Sciences, Psychology and Psychiatry Unit, University of Florence, via di San Salvi 12, 50100 Florence, Italy.

^3^ Department of Biomedical Experimental and Clinical Sciences, SOD Diabetology, University of Florence and Careggi University Hospital, Florence. Italy.

^4^ Diabetology, University of Florence and Careggi Teaching Hospital, Largo Brambilla 3, 50134, Florence, Italy.

^5^ Psychiatry Unit, University of Florence and Careggi Teaching Hospital, Largo Brambilla 3, 50134, Florence, Italy.

Corresponding author: Dr. Emanuele Cassioli, Psychiatry Unit, Department of Health Sciences, University of Florence, Largo Brambilla 3, 50134, Florence, Italy. Email: emanuele.cassioli@unifi.it

###### FEED (Florence Emotional Eating Drive)

We all respond to different emotions with different behaviors. Some emotions may trigger in ourself the desire to eat or make us feel hungry.

Please indicate how often you happen to feel the emotions reported below and the extent of how much such emotions drive you to eat, or desire to.

By answering the following questions you will contribute to the validation of the FEED questionnaire. Your data will be treated as strictly confidential.

Please report the informations required in the fields below. Your data will be used to create a personal ID for proper data management.

Thank you for your cooperation.

Sex: M □ F □

Age:

Education:

Marital status:

Your mother’s initials:

The last three figures of your mobile phone number:

|  | How often do you feel…? | | | | | How strong is your drive for eating when you feel…? | | | | |
| --- | --- | --- | --- | --- | --- | --- | --- | --- | --- | --- |
|  | *Never* | *A few times* | *Sometimes* | Often | *Always* | *No desire to eat* | *A small desire to eat* | *A moderate desire to eat* | A strong urge to eat | *An overwhelming urge to eat* |
| Resentful |  |  |  |  |  |  |  |  |  |  |
| Discouraged |  |  |  |  |  |  |  |  |  |  |
| Shaky |  |  |  |  |  |  |  |  |  |  |
| Worn out |  |  |  |  |  |  |  |  |  |  |
| Inadequate |  |  |  |  |  |  |  |  |  |  |
| Rebellious |  |  |  |  |  |  |  |  |  |  |
| Blue |  |  |  |  |  |  |  |  |  |  |
| Jittery |  |  |  |  |  |  |  |  |  |  |
| Sad |  |  |  |  |  |  |  |  |  |  |
| Uneasy |  |  |  |  |  |  |  |  |  |  |
| Irritated |  |  |  |  |  |  |  |  |  |  |
| Jealous |  |  |  |  |  |  |  |  |  |  |
| Worried |  |  |  |  |  |  |  |  |  |  |
| Frustrated |  |  |  |  |  |  |  |  |  |  |
| Lonely |  |  |  |  |  |  |  |  |  |  |
| Furious |  |  |  |  |  |  |  |  |  |  |
| On edge |  |  |  |  |  |  |  |  |  |  |
| Confused |  |  |  |  |  |  |  |  |  |  |
| Nervous |  |  |  |  |  |  |  |  |  |  |
| Angry |  |  |  |  |  |  |  |  |  |  |
| Guilty |  |  |  |  |  |  |  |  |  |  |
| Bored |  |  |  |  |  |  |  |  |  |  |
| Upset |  |  |  |  |  |  |  |  |  |  |

**Table 1 of Supplementary Material – FEED scoring table**

|  |  | **DRIVE FOR EATING** | | | | |
| --- | --- | --- | --- | --- | --- | --- |
|  |  | **0** | **1** | **2** | **3** | **4** |
| **FREQUENCY OF EMOTION** | **0** | 0 | 0 | 0 | 0 | 0 |
|  | **1** | 0 | 1 | 2 | 3 | 4 |
|  | **2** | 0 | 2 | 4 | 5 | 6 |
|  | **3** | 0 | 3 | 5 | 7 | 8 |
|  | **4** | 0 | 4 | 6 | 8 | 9 |
